# Acneiform type of mogamulizumab‐associated rash

**DOI:** 10.1111/ddg.15816

**Published:** 2025-07-02

**Authors:** Inga Hansen‐Abeck, Glenn Geidel, Finn Abeck, Anne Menz, Stefan W. Schneider, Nina Booken

**Affiliations:** ^1^ Department for Dermatology and Venerology University Medical Center Hamburg‐Eppendorf Hamburg Germany; ^2^ Institute of Pathology University Medical Center Hamburg‐Eppendorf Hamburg Germany

**Keywords:** cutaneous t‐cell lymphoma, mogamulizumab‐associated rash, mogamulizumab, Mycosis fungoides, Sézary syndrome

Dear Editors,

Mogamulizumab‐associated rash (MAR) is one of the most common adverse events (AE) of the anti‐C‐C chemokine receptor type 4 (CCR4) antibody mogamulizumab, which is approved as a second‐line therapy for the treatment of mycosis fungoides (MF) and Sézary syndrome (SS).^1^ In the pivotal study (MAVORIC study), the prevalence of MAR was reported with 24% and was described as the most common AE leading to discontinuation.^1^

Many different types of MAR have been described with four clinically predominant patterns: folliculotropic MF‐like scalp plaques with alopecia, papules and/or plaques, photodermatitis, and morbilliform/erythrodermic dermatitis.^2^, ^3^ In addition, individual cases of rare types such as palmoplantar hyperkeratosis or imitation of lupus miliaris disseminatus faciei have been described recently.^3^, ^4^, ^5^, ^6^ Regarding histological reaction patterns, three major patterns of MAR have been identified with mixed forms being common: spongiotic/psoriasiform dermatitis, interface/lichenoid dermatitis, and granulomatous dermatitis.^3^, ^7^

We report the case of a 42‐year‐old male patient with stage IIB (T3 N0 M0 B0) folliculotropic MF with large‐cell transformation. After initial therapy with bexarotene, we started treatment with mogamulizumab due to disease progression (modified Severity Assessment Tool [mSWAT] 56). Mogamulizumab was administered following premedication with clemastine and paracetamol. No other medication was given, in particular no glucocorticosteroids or antibiotics. After five cycles of mogamulizumab (1 mg/kg bodyweight), the patient reported new skin changes, whereas the tumor nodules and plaques showed marked regression (Figure 1a–c). Clinically, he presented with follicular pustules on the face, trunk, arms and legs, consistent with acneiform dermatitis. There was no maculopapular rash, blistering, or epidermolysis, allowing clinical exclusion of drug reactions such as DRESS syndrome or toxic epidermal necrolysis. Skin samples were taken and showed pustular folliculitis and perifolliculitis with perivascular lymphocytic infiltrates with a high proportion of CD8‐positive cells. The acute inflammatory and histiocyte‐rich reaction with a strong CD8‐positive component and the polyclonal T‐cell receptor rearrangement was consistent with MAR (Figure 2). Due to the multilocular distribution and the clinical presentation with follicular pustules, the skin findings did not correspond to previously described types of MAR, leading to the first diagnosis of acneiform MAR. Therefore, we initiated treatment with topical steroids and topical antibiotics while continuing therapy with mogamulizumab. As there was no improvement of MAR, we started additional treatment with doxycycline 200 mg per day. Two weeks after initiating doxycycline, the patient reported a significant reduction in skin changes and pruritus. In the further course, a brief attempt to discontinue doxycycline resulted in a flare‐up of MAR. Doxycycline was therefore continued, as well as mogamulizumab. Mycosis fungoides remains well controlled with mSWAT of 16 after ten cycles of mogamulizumab (Figure 1e–f). No other mogamulizumab‐related AEs have occurred in this patient.

**FIGURE 1:**
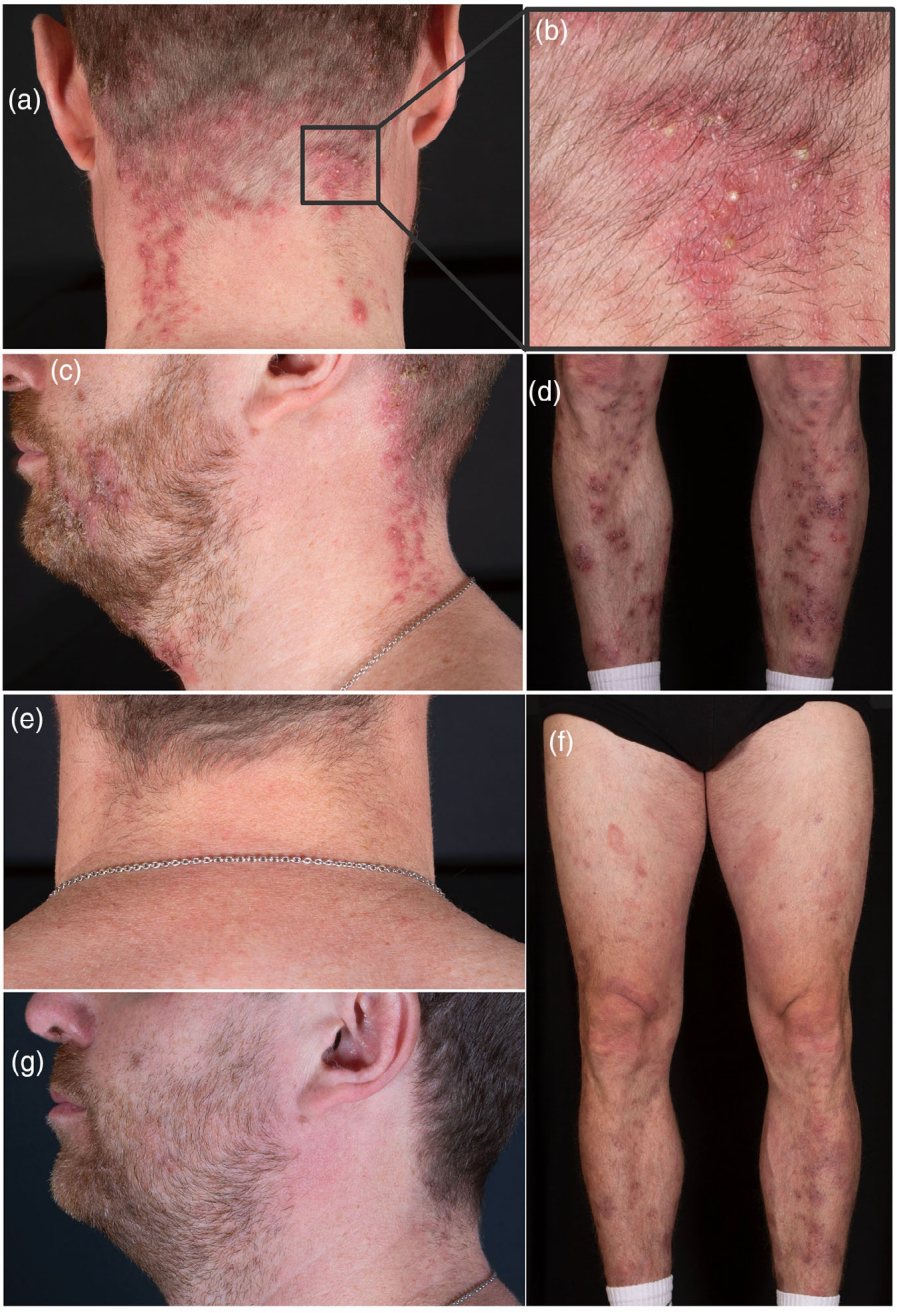
Clinical presentation of acneiform mogamulizumab‐associated rash with erythematous papules and pustules on the (a) neck, (b) pustules in magnification, (c) legs, and (d) face. (e) Neck, (f) legs, and (g) face after 8 weeks of treatment with doxycycline.

**FIGURE 2:**
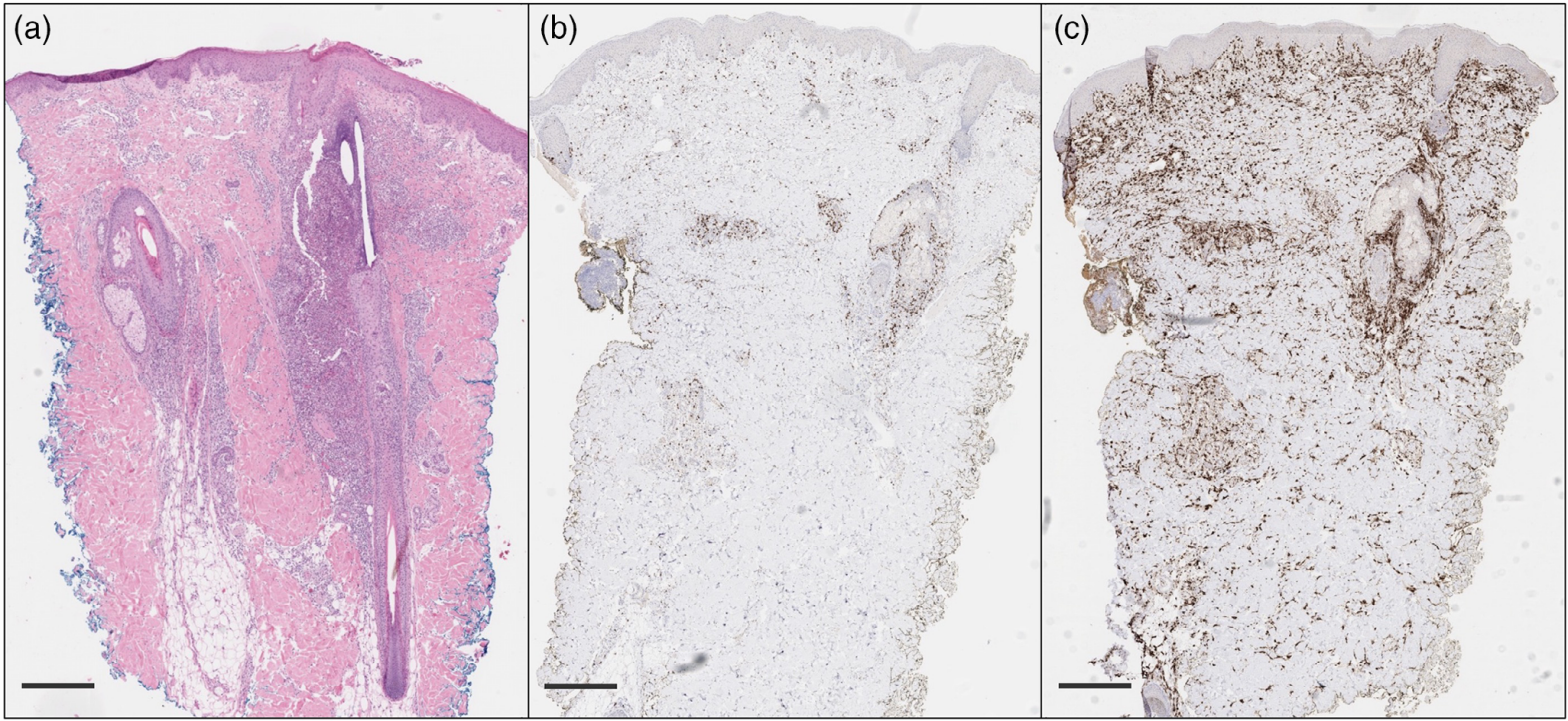
Histological features of a mogamulizumab‐associated rash with neutrophil‐ and histiocyte‐rich follicular inflammation and a high proportion of CD8⁺ cells (scale bar: 500 µm). (a) Hematoxylin‐eosin staining reveals acute folliculitis and perifolliculitis with perivascular lymphocytic infiltrates, along with neutrophil‐ and histiocyte‐rich inflammation of the hair follicles. (b) Immunohistochemistry shows a strong CD8⁺ component with a CD4:CD8 ratio of 2:1. (c) CD163 staining demonstrates a marked proportion of histiocytic cells.

To our knowledge, this is the first report of an acneiform MAR. To date, only one case of MAR with occurring of pustules has been reported with a pustular eruption on the scalp, which was histologically diagnosed as an eosinophilic folliculitis.^8^ Acneiform rash is a well‐known AE of treatment with EGFR‐inhibitors.^9^

The pathophysiological background of MAR is not yet fully understood, but one possible explanation is the decrease in CCR4‐expressing regulatory T‐cells, which leads to the activation of cytotoxic CD8^+^ T‐lymphocytes.^10^ Cases with follicular involvement of lymphocytes in MAR have been reported, ranging from mild forms to follicular destruction of the hair follicle.^7^ However, the additional occurrence of pustules cannot yet be explained. We assume that neutrophil chemotaxis may be triggered by severe folliculitis.

Given the growing evidence that the presence of MAR is associated with improved treatment response, our patient exhibited a rapid and sustained clinical response, achieving partial remission. Management of MAR is depending on the severity and its impact on quality of life. The use of topical or systemic glucocorticosteroids, symptomatic therapy with antipruritic medications (e.g., antihistamines), and, if necessary, the addition of methotrexate is recommended.^11^ The discontinuation of mogamulizumab should be evaluated based on the severity of the MAR.^6^, ^11^ In this case, we continued treatment with mogamulizumab despite the development of MAR grade III (> 30% of the body surface) because of the patient's good quality of life. It is also the first report of effective treatment of MAR with doxycycline.

In conclusion, MAR can manifest in various clinical and histological forms, but it may be difficult to distinguish from the underlying disease.^3^, ^6^ Current data suggest that MAR is associated with a significantly improved treatment response, highlighting the importance of distinguishing it from disease progression to avoid potentially premature discontinuation of therapy.^3^, ^6^

## CONFLICT OF INTEREST STATEMENT

N.B. received honoraria for presentations and support for attending meetings and/or travel from Kyowa Kirin. I.H.‐A. received support for attending meetings and/or travel from Kyowa Kirin.
